# Osteopenic Effect of Disulfiram Therapy in Patients With Alcohol Use Disorder

**DOI:** 10.7759/cureus.73643

**Published:** 2024-11-13

**Authors:** Soma Saha, Monika Mittal, Ravinder Goswami, Parul Narang, Yatan Pal Singh Balhara, Naibedya Chattopadhyay

**Affiliations:** 1 Department of Endocrinology, All India Institute of Medical Sciences, New Delhi, New Delhi, IND; 2 Department of Endocrinology, Council of Scientific and Industrial Research-Central Drug Research Institute, Lucknow, IND; 3 Department of Psychiatry, All India Institute of Medical Sciences, New Delhi, New Delhi, IND

**Keywords:** alcohol use disorder, bone mineral density, bone turnover markers, disulfiram, therapeutic adherence

## Abstract

Background: Disulfiram is widely used to treat alcohol use disorder. Alcohol per se adversely affects bone health. In the experimental study, disulfiram leads to apoptosis of osteoblast and significant osteopenia in adult rats. The present study explored the possibility of the occurrence of similar osteopenic effects of disulfiram therapy in patients with alcohol use disorder.

Aim: To assess the impact of disulfiram on bone health in patients with alcohol use disorder.

Design and methods: The study design was longitudinal observational. Subjects included 40 males with alcohol use disorder (aged 35.7±7.1 years, body mass index: 23.4±3.8 kg/m^2^) who were initiated on disulfiram therapy at our tertiary care addictive disorders treatment center. They were assessed for bone mineral density at the lumbar spine, total hip, femoral neck, and forearm, at baseline and again at three time points (i.e., three-, six-, and 12-month follow-up on disulfiram therapy). Since all the patients did not come at all the above three follow-up points, the follow-up on the maximum period of disulfiram therapy at any of these three time points was considered as the final follow-up. Serum total-calcium, phosphorus, albumin, intact parathyroid hormone (iPTH), 25-hydroxy vitamin D (25(OH)D), and bone-turnover marker (i.e., osteocalcin and β-carboxy-terminal telopeptides (β-CTx)) were measured at baseline and final follow-up. Statistical analyses were performed using Student’s t-test for normally distributed and the Mann-Whitney U test for non-normally distributed data. A two-tailed p-value <0.05 was considered significant.

Results: The median (interquartile range) duration of alcohol use and alcohol use disorder were 15.0 (10.0-20.0) and 8.0 (5.0-12.0) years, respectively. Patients had taken disulfiram for a mean period of 225±109 days, with 65% therapeutic adherence. Serum transaminases improved significantly during follow-up, indicating abstinence from alcohol intake. Despite alcohol withdrawal, lumbar spine bone mineral density decreased significantly at the final follow-up (0.942±0.105 gm/cm^2 ^vs 0.924±0.108 gm/cm^2^, p=0.003). A decrease in lumbar-spine bone mineral density > least significant change occurred in 41.7% of patients following 12 months of disulfiram use. A reduction in bone mineral density correlated with adherence to disulfiram therapy (r=0.233, p=0.15). The median serum β-CTx/osteocalcin ratio increased after disulfiram (12.5 (9.7-17.4) vs 16.3 (11.8-19.9), p=0.033).

Conclusions: This study highlights a significant reduction in lumbar-spine bone mineral density and an increase in the β-CTx/osteocalcin ratio in patients with alcohol use disorder undergoing disulfiram therapy. These findings suggest that, despite the benefits of alcohol cessation facilitated by disulfiram, there is a notable risk of osteopenia that warrants careful monitoring. Clinicians should consider regular bone health assessments and proactive measures to mitigate bone density loss in this patient population.

## Introduction

Alcohol use disorder affects various systems, including gastrointestinal, cardiovascular, and central/peripheral nervous systems [1,2]. There is limited information on the impact of alcohol use on bone health and changes following the cessation of alcohol during disulfiram therapy [3-10]. Alcohol use disorder can influence bone mineral density, either decreasing or increasing it, depending on factors such as alcohol quantity, duration, smoking, malnutrition, and more [4-10]. Our recent study observed a high prevalence of thoracic vertebral fractures in patients with long-standing alcohol use disorder, likely due to frequent falls rather than osteoporosis [3]. Disulfiram is a common treatment for alcohol use disorder [11]. It causes severe physical discomfort during alcohol intake by inhibiting aldehyde dehydrogenase and increasing serum acetaldehyde levels, encouraging alcohol abstinence. However, experimental data have shown that disulfiram can enhance osteoblast apoptosis, leading to significant osteopenia [12]. The present study was conducted to explore the possibility of the occurrence of similar osteopenic effects of disulfiram therapy in patients with alcohol use disorder.

## Materials and methods

The study design was longitudinal and observational. Subjects were patients with alcohol use disorder attending the addictive disorders treatment clinic, AIIMS, New Delhi, during 2019-2020. Alcohol use disorder was defined as, per the Diagnostic and Statistical Manual of Mental Disorders (DSM-5) criteria, a problematic pattern of alcohol use, leading to clinically significant impairment or distress [13]. The essential inclusion criterion for study participants was the use of disulfiram for alcohol use disorder and a minimum follow-up period of three months on disulfiram therapy. The three-month follow-up was included in this study to allow an early assessment of the impact of disulfiram therapy on bone mineral density. This early follow-up was also considered necessary to ensure sufficient study subjects following disulfiram therapy in view of the high probability of dropouts during follow-up in alcohol use disorder. The information on the nature of alcoholic beverages used, their duration, and the amount consumed were recorded for each patient through detailed clinical history. Patients dependent on other psychoactive substances, except nicotine, or on anti-convulsant, steroid, or anti-tubercular therapy during the last six months, were excluded to minimize their confounding effect on areal bone mineral density. All the patients were assessed for bone mineral density at the lumbar spine, total hip, femoral neck, and forearm, at baseline and again at three time points (i.e., three-, six-, and 12-month follow-up on disulfiram therapy). Since all the patients did not come at all the above three follow-up points, the follow-up on the maximum period of disulfiram therapy at any of these three time points was considered as their final follow-up.

After informed consent, each patient was prescribed an oral 250 mg of disulfiram once a day. Disulfiram tablets were provided free of cost with monthly refilling at the deaddiction clinics to decrease the financial burden and promote adherence to disulfiram therapy. Patients' adherence to disulfiram therapy was monitored at each visit through self-reporting, caregiver feedback, and pill counts. Therapeutic adherence was calculated as the percentage of the actual number of tablets consumed and the expected number of tablets to be taken during the follow-up interval. All patients also attended sessions on relapse prevention and were counseled for lifestyle changes as part of standard care along with disulfiram therapy.

Baseline bone mineral density was measured using dual-energy X-ray absorptiometry (Discovery A 84023; Hologic Inc., MA) one week before starting disulfiram therapy and during follow-ups at three, six, and 12 months on disulfiram therapy [14]. Bone mineral density was defined as bone mineral content per unit area (g/cm^2^). Bone mineral density was measured at the lumbar spine (L1-L4), hip, and forearm, as per the International Society for Clinical Densitometry (ISCD) guidelines [9]. The bone mineral density T-score lumbar spine > -1.0, -1.0- <-2.5, and <= -2.5 were considered as normal, osteopenia, and osteoporosis as per WHO criteria, respectively [15]. The coefficient of variation (CV) of the precision error was calculated as per the ISCD guidelines [16]. The CV at the lumbar spine, hip, femoral neck, and total forearm was 0.69%, 1.73%, 1.40%, and 1.12%, respectively, and CV for the least significant change (LSC) at these corresponding sites was 1.92%, 4.78%, 3.88%, and 3.11%, respectively [14]. Lumbar vertebrae with fractures were excluded from the calculation of bone mineral density.

The trabecular bone score was assessed along with bone mineral density using iNsight software (v3.0.2.0; Med-Imaps, Bordeaux, France). The trabecular bone score is a recently developed non-invasive tool that analyzes the gray-level texture using pixels on lumbar spine dual X-ray absorptiometry images and thereby captures information related to trabecular microarchitecture. Bone microarchitecture was classified as degraded, partially degraded, and normal as defined by McCloskey et al. with a TBS >1.310 indicating dense good microarchitecture connectivity and a score < 1.230 indicating "degraded" microarchitecture susceptible to vertebral fractures [17]. The CV of the precision error for TBS was 1.39%, with the LSC being 3.85% [14].

Biochemical investigations

Various biochemical parameters were measured at each follow-up visit. Blood samples were collected after an overnight fast. The serum was separated by centrifugation at 4℃ and stored in multiple aliquots at -20℃. All the investigations were carried out in the endocrine service laboratories of our institute. The serum total calcium, inorganic phosphate, and alkaline phosphatase were measured on Cobas C111auto-analyzer (normal: 2.13-2.63 mmol/L and 0.81-1.45 mmol/L and 80-240 IU/L, respectively; Roche, Germany). Their intra-assay and inter-assay coefficients of variation were 3.5-5.0%. Serum 25(OH)D was measured by chemiluminescence (LIAISON, DiaSorin, Inc., MN). Serum intact PTH (normal: 15-65 ng/L), osteocalcin (both N-mid and intact), and β-CTx (all type 1 collagen-degradation fragments) were measured using electrochemiluminescence Cobas e411 (Roche). The performance of osteocalcin and β-CTx was checked by Precicontrol Varia level 1 (range: 13.9-22.7 ng/mL and 197-343 pg/mL) and level 2 controls (range: 74.1-121 ng/mL and 526-914 pg/mL).

Statistical analysis

Data are presented as mean and standard deviation (SD), median with interquartile range (IQR), and percentages. Student's t-test was performed for inter-group comparison of normally distributed data. The Mann-Whitney U test was used for parameters that were not normally distributed. Fisher's exact test was used to assess differences in the frequency of various parameters. All analyses were performed using SPSS (version 20.0; IBM, Armonk, NY). A two-tailed p-value <0.05 was considered significant.

The study protocol was approved by the Institute Ethics Committee at AIIMS, New Delhi (IEC-441/04.08.2017, RP-17/2017).

## Results

**Figure 1:**
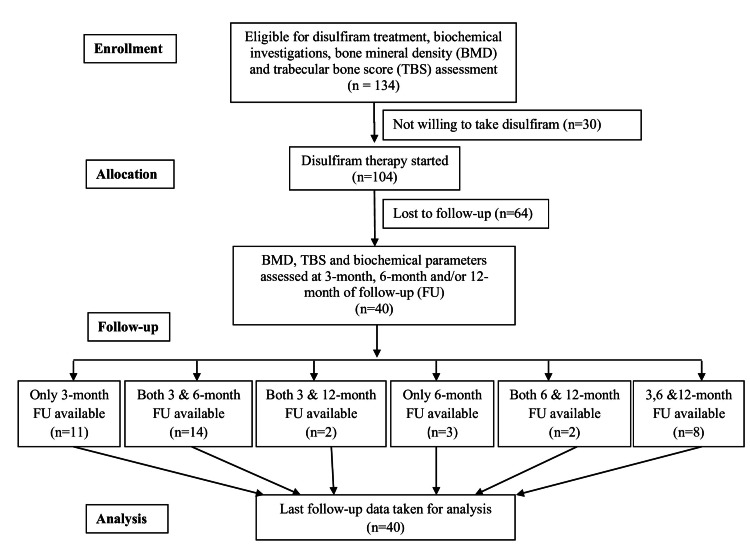
Flow of the patients and number of patients followed up at different visits

The study included 40 male patients with alcohol use disorder. Figure 1 shows the flow of 40 patients and their follow-up at various intervals.

Their baseline demographic and biochemical parameters are shown in Table 1. Their mean age and BMI were 35.7±7.1 years and 23.4±3.8 kg/m^2^, respectively. The median (IQR) duration of alcohol use and AUD were 15.0 (10.0-20.0) years and 8.0 (5.0-12.0) years, respectively. All patients included in this study were males as females with AUD uncommonly attended the deaddiction clinic. There was a history of concomitant tobacco use in 38 subjects (95%). The average duration and adherence to disulfiram therapy were 225±109 days and 65.0±27.9%, respectively. Out of the 40 patients, 35 patients completed the three months, 27 patients completed six months, and 12 patients completed 12 months of follow-up after disulfiram therapy.

**Table 1:** Clinical and biochemical characteristics of 40 patients with alcohol use disorder (AUD) on disulfiram therapy BMI, body mass index; SGOT, serum glutamic oxaloacetic transaminase; SGPT, serum glutamic-pyruvic transaminase; iPTH, intact parathyroid hormone; 25(OH)D, 25-hydroxyvitamin D

Parameters	AUD patients on disulfiram for all or at least one follow-up during one year (n=40)
Age (years)	35.7 ± 7.1
Body Mass Index (kg/m^2^)	23.4 ± 3.8
Duration of alcohol use (years)	15.0 (10.0-20.0)
Duration of AUD (years)	8.0 (5.0-12.0)
Absolute alcohol intake (g/day)	193.0 (109.0-257.0)
Severity of AUD (years)	8.3 ± 1.2
Tobacco intake (n,%)	38/40 (95%)
Serum total calcium (mmol/L)	2.35 ± 0.15
Serum phosphate (mmol/L)	1.29 ± 0.36
Serum albumin (g/L)	47.0 ± 4.8
SGOT (IU)	48.0 (39.0-94.0)
SGPT (IU)	55.0 (35.0-96.0)
Serum iPTH (ng/L)	52.25 ± 23.65
Serum 25(OH)D (nmol/L)	27.1 (18.0-39.3)

Change in bone mineral density and bone markers after disulfiram therapy

Table 2 shows the change in biochemical parameters, including bone-related parameters before and after disulfiram therapy. There was a significant reduction in serum transaminase (i.e., glutamic-pyruvic transaminase and serum glutamic oxaloacetic transaminase during follow-up). In contrast, there was a significant decrease in lumbar spine bone mineral density after an average of 225 days on disulfiram therapy (0.942±0.105 gm/cm^2^ vs 0.924±0.108 gm/cm^2^, p=0.003). A similar pattern of significant decrease in bone mineral density at the lumbar spine was observed at three, six, and 12 months of disulfiram therapy. However, there was no significant fall in the bone mineral density at the hip, femoral neck, and forearm (ultra-distal and mid and proximal radius) after disulfiram therapy.

**Table 2:** Comparison of biochemical and bone densitometry parameters in patients with alcohol use disorder (AUD) before and after disulfiram therapy (n=40) iPTH, intact parathyroid hormone; 25(OH)D, 25-hydroxyvitamin D SGOT, serum glutamic oxaloacetic transaminase; SGPT, serum glutamic-pyruvic transaminase; CTx, beta-isomerised carboxy-terminal cross-linking telopeptide

Characteristic	Disulfiram treatment		p
	Before	After	
Serum calcium (mmol/L)	2.35 ± 0.15	2.35 ± 0.10	0.45
Serum phosphorus (mmol/L)	1.29 ± 0.36	1.20 ± 0.23	0.20
Serum alkaline phosphatise (IU/L)	247 ± 75	261 ± 72	0.17
Serum 25(OH)D (nmol/L)Median (IQR)	25.0 (17.5-39.7)	21.2 (15.5-37.4)	0.17
Serum iPTH (ng/L)	51.3 ± 23.4	63.6 ± 30.8	0.08
Serum total protein (g/L)	75.0 ± 6.0	75.0 ± 3.0	0.79
Serum albumin (g/L)	48.0 ± 6.0	47.0 ± 4.0	0.64
Serum bilirubin total (µmol/L)	10.3 ± 5.1	10.3 ± 5.1	0.47
SGOT (U/L) Median (IQR)	48.0 (39.0-100.0)	29.0 (23.0-51.0)	0.001
SGPT (U/L) Median (IQR)	61.0 (36.5-101.5)	33.0 (24.5-57.0)	0.001
Bone Mineral Density (g/cm^2^)			
Lumbar_1_	0.900 ± 0.093	0.876 ± 0.092	0.003
Lumbar_2_	0.945 ± 0.107	0.926 ± 0.106	0.005
Lumbar_3_	0.971 ± 0.124	0.964 ± 0.123	0.25
Lumbar_4_	0.947 ± 0.115	0.928 ± 0.129	0.008
Lumbar_1-4_	0.942 ± 0.105	0.924 ± 0.108	0.003
Femoral neck	0.763 ± 0.097	0.758 ± 0.097	0.20
Femoral trochanter	0.624 ± 0.090	0.629 ± 0.094	0.52
Femoral Inter-trochanter	1.057 ± 0.132	1.049 ± 0.139	0.25
Total hip	0.876 ± 0.111	0.871 ± 0.113	0.37
Forearm ultra-distal	0.465 ± 0.057	0.459 ± 0.057	0.07
Forearm mid	0.658 ± 0.051	0.657 ± 0.054	0.73
Forearm 1/3	0.774 ± 0.058	0.773 ± 0.061	0.59
Forearm total	0.628 ± 0.048	0.626 ± 0.050	0.41
Bone mineral content lumbar spine	55.83 ± 14.46	55.02 ± 11.34	0.60
Trabecular bone score	1.348 ± 0.076	1.347 ± 0.084	0.91
Bone turnover markers			
Serum osteocalcin (ng/mL) median (IQR)	11.11 (8.50-16.89)	18.95 (12.77-22.81)	0.007
Serum CTx (pg/mL) median (IQR)	184 (120-224)	256 (208-467)	<0.001
CTx/Osteocalcin (pg/ng) median (IQR)	12.5 (9.7-17.4)	16.2 (11.8-19.9)	0.033

**Figure 2:**
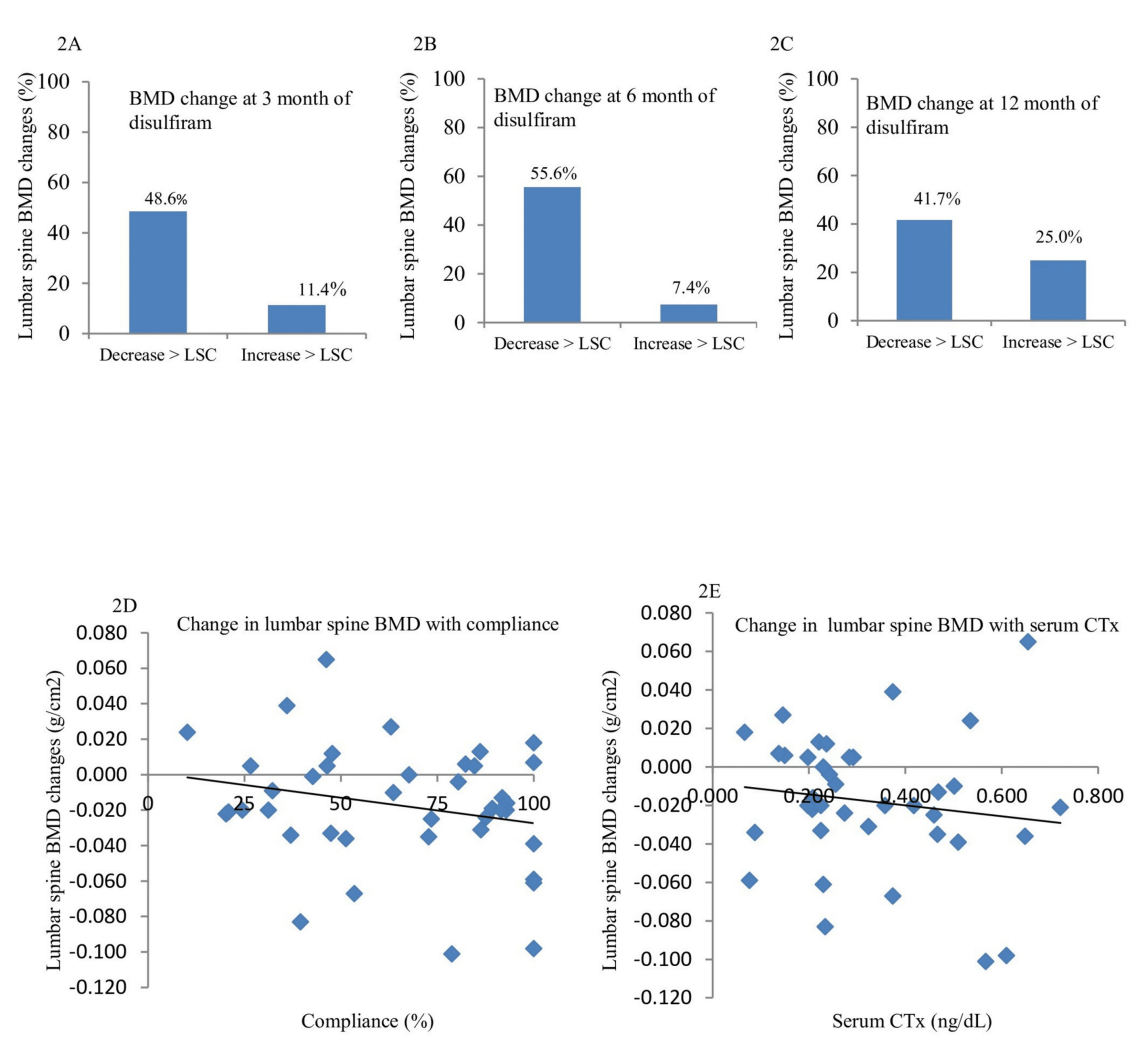
Percentage of patients showing the change in lumbar spine bone mineral density (BMD) above and below the least significant change (LSC) at the three months (A), six months (B), and 12 months (C) of follow-up after disulfiram therapy The scatter plot showing the delta change in lumbar spine BMD at the final follow-up with disulfiram compliance (D) and serum β-CTx (E).

At three months of disulfiram use, bone mineral density decreased to LSC in 48.6% (Figure 2A, Table 3). A similar decrease in bone mineral density of 55.6% and 41.7% was observed at six and 12 months of disulfiram therapy, respectively (Figures 2B, 2C, Table 3). The change in lumbar spine bone mineral density from baseline to the last follow-up showed a trend of direct correlation with the disulfiram compliance (r=0.233, p=0.15; Figure 2D). The mean trabecular bone score, serum total calcium, phosphate, alkaline phosphatase, 25(OH)D, and iPTH did not show significant change after disulfiram therapy (Table 2).

**Table 3:** Percentage of patients with alcohol use disorder showing the change in bone mineral density above and below the least significant change during follow-up after disulfiram therapy %, percentage

Follow-up interval	Decrease > least significant change	Increase > least significant change
3 months (n = 35)	48.6%	11.4%
6 months (n = 27)	55.6%	7.4%
12 months (n = 12)	41.7%	25%

Median serum β-CTx (184 (120-224) pg/mL vs 256 (208-467) pg/mL) and serum osteocalcin (11.11 (8.50-16.89) ng/mL vs 18.95 (12.77-22.81) ng/mL) increased significantly after disulfiram therapy (p<0.001 and 0.007, respectively; Table 2). The β-CTx/osteocalcin ratio (pg/ng) increased from 12.5 (9.7-17.4) to 16.3 (11.8-19.9) after disulfiram therapy (p=0.03). The serum β-CTx at the final follow-up showed a trend of direct correlation with the change in lumbar bone mineral density (r=0.142, p=0.39; Figure 2E).

## Discussion

The present study investigated the detrimental effect of disulfiram therapy on bone health in patients with alcohol use disorder. This was considered necessary in view of the findings observed in in-vitro experimental studies on disulfiram from our center and by other investigators [12,18,19]. Our earlier experimental study indicated a decreased osteoblastic activity and trabecular bone volume and osteopenia following oral disulfiram administration at a human equivalent dose in rats [12].

The present study showed that there was a significant fall in lumbar spine bone mineral density after an average of eight months of disulfiram therapy. Interestingly, the fall in bone mineral density was evident even at three months after disulfiram therapy (i.e., at the first follow-up), which is akin to a rapid decline in bone health observed within four weeks in experimental rats. The early fall in bone mineral density could be due to the effect of disulfiram use or could also be due to continued use of alcohol. However, a significant improvement in liver transaminases at all follow-ups indicated adequate abstinence from alcohol following disulfiram therapy. This fact suggests the primary role of disulfiram in the deterioration of bone health in humans akin to our earlier pre-clinical experimental study [12]. The adverse effect of disulfiram on bone health was supported by several other observations. Firstly, the significant decrease in bone mineral density at the lumbar spine rather than the hip following disulfiram therapy is similar to the adverse resorptive effect of excessive steroid or thyroxine therapy. The positive relationship between delta fall at bone mineral density of the lumbar spine and disulfiram compliance also indicated an association between disulfiram and deterioration in bone health.

Further, a significant rise in the β-CTx/osteocalcin ratio following disulfiram therapy indicated that the increase in bone resorption was not sufficiently coupled by formation. As expected, the initial manifestation of increased resorption was observed first in the trabecular-rich lumbar spine rather than in the cortical-rich compact hip bone. Changes in bone markers along with bone mineral density highlighted the mechanistic aspect related to the adverse effect of disulfiram on bone health. These observations also suggest a possible beneficial effect of using anti-osteoporotic medicine such as bisphosphonates as an adjuvant therapy along with disulfiram therapy to prevent deterioration in bone health.

Though the present study has the strength of being the first clinical study investigating the effect of disulfiram on bone health, it has limitations of not including females with alcohol use disorder, and high drop-outs despite diligent efforts by the investigators. Though high drop-outs are observed in most addictive disorders, it was also attributed to the COVID-19 epidemic, which occurred during the study period [20,21]. Thus, our study provides the first clue of the possible detrimental effect of disulfiram therapy on bone health in alcohol use disorder. Further studies are required to support our observation in different populations.

## Conclusions

This study highlights a significant reduction in lumbar-spine bone mineral density and an increase in the β-CTx/osteocalcin ratio in patients with alcohol use disorder on disulfiram therapy. This is the first study demonstrating the osteopenic effect of disulfiram on bone in patients with alcohol use disorder. These findings suggest that, despite the benefits of alcohol cessation facilitated by disulfiram, there is a notable risk of osteopenia that warrants careful monitoring. Clinicians should consider regular bone health assessments and proactive measures to mitigate bone density loss in this patient population. Further research is essential to explore preventive strategies using anti-osteoporotic therapy and optimize treatment protocols, ensuring the effective management of bone health in alcohol use disorder.
